# China’s outward foreign direct investment in energy sector: The role of “intimate” relations between countries

**DOI:** 10.1371/journal.pone.0254199

**Published:** 2021-07-12

**Authors:** Na Tan, Liang Chang, Rui Guo

**Affiliations:** 1 Research Center for International Trade and Economics, Guangdong University of Foreign Studies, Guangzhou, China; 2 School of Accounting, Guangdong University of Foreign Studies, Guangzhou, China; 3 Research Center for Cross-Border M&A and Innovation Strategy, Guangdong University of Foreign Studies, Guangzhou, China; 4 School of Finance, Guangdong University of Foreign Studies, Guangzhou, China; Taiyuan University of Science and Technology, CHINA

## Abstract

Based on the data of China’s outward foreign direct investment (OFDI) in energy sector to 133 countries from 2005 to 2014, this paper uses a gravity model to investigate the impact of “intimate” relations on China’s OFDI locations in energy sector. We find that the “intimate” relations have significant effects on China’s OFDI locations in energy sector, namely: bilateral senior leaders’ visits, institutional distance, genetic distance, and immigration. Holding other factors fixed, for each one more bilateral senior leaders’ visit between China and the host country, China’s OFDI in energy sector for the host country will increase by 5.44%. If the genetic distance from China and host country increases by 1%, China’s OFDI in energy sector will fall by 1.69%. For every 1% increase in the institutional distance between China and host country, China’s energy OFDI will decrease by 1.09%. For every 1% increase in a country’s immigration to China, China’s energy OFDI will increase by 0.46%. Further, after distinguishing developed and developing countries, we find that compared with developed countries, “intimate” relations have greater impacts on China’s energy OFDI in developing countries. Finally, based on the dominance analysis, considering China’s “intimate” relations with countries along the “Belt and Road” and current locations of China’s OFDI, we find that China should further expand energy investment in countries along the “Belt and Road”.

## I. Introduction

The impact of political and cultural factors on energy investment flows across countries is important for a country’s energy security [1–3]. China has implemented the “One Belt One Road” initiative since 2013, and one important policy of this initiative is to strengthen bilateral and multilateral energy cooperation with countries along the “Belt and Road”. Therefore, studying the impact of political and cultural factors such as “intimate” relations on energy investment between China and other countries is valuable for international energy investment and energy security.

**Fig 1:**
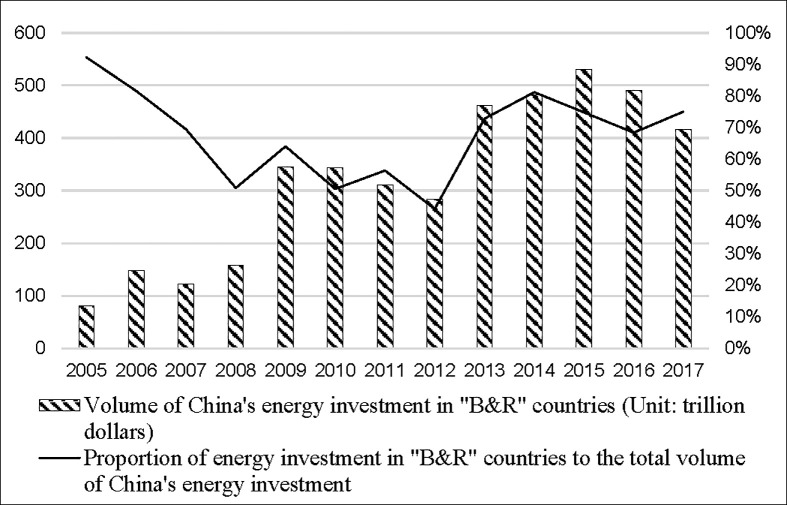
China’s OFDI in energy sector to “B&R” countries from 2005 to 2017. Fig 1 shows the investment of Chinese energy companies in “B&R” countries from 2005 to 2017. The investment covers petroleum, hydropower, alternative energy, natural gas and coal. The data is available from the China Global Investment Tracker database released by the American Enterprise Institute and the Heritage Foundation.

In recent years, with the advancement of the “Belt and Road” initiative, China’s outward foreign direct investment (OFDI for short) in energy sector has grown rapidly [4]. Especially the direct investment stocks of Chinese energy companies in the countries along the “Belt and Road” (“B&R” countries for short) have shown a steady upward trend. Fig 1 shows the investment of Chinese energy companies in “B&R” countries from 2005 to 2017. The amount of investment by Chinese energy companies in the “B&R” countries has shown a clear upward trend since 2005, rising from $8.1 billion in 2005 to $41.61 billion in 2017, with a significant increase of about 5.1 times in 12 years.

The rapid growth of overseas investment in energy industry is mainly due to international energy security and the shortage of domestic energy. From the domestic perspective, energy consumption and economic growth usually have a strong consistency in the trend [5]. With the rapid economic development of China, the gap between domestic energy production and consumption has widened year by year. Therefore, the external dependence of energy has been increasing. As China’s economic growth has declined in recent years, the growth in energy sector demand has also slowed down. Still, the growth rate as 1.5% of energy consumption is the highest among the world, and China has maintained the position of the largest incremental energy market in the world for 15 years consecutively, which is released by the BP Statistical Review of World Energy (2018). From the international perspective, the current global price fluctuations of energy products have been intensified due to the unstable international situation, which leads to not only higher production costs and commercial risks but also more potential risks in politics and energy security [6, 7]. In such an impoverished environment, OFDI in energy sector has become an important way for China to explore foreign energy markets.

However, it is worth noting that the growth of China’s investment in energy sector in “B&R” countries has slowed down since 2013. The proportion of China’s energy investment in “B&R” countries to the total volume of China’s energy investment has decreased from 92.26% in 2005 to 75.07% in 2017, even fell to 44.19% in 2012, which means that China’s energy investment in “B&R” countries may be inadequate.

The “Belt and Road” countries in central Asia, west Asia and north Africa are rich of energy resources. The data from China National Petroleum Corporation (CNPC) Economics & Technology Research Institute shows that the remaining proven oil reserves in “B&R” countries are 133.8 billion tons, accounting for 57% of the global reserves. The remaining proven natural gas reserves reach 155 trillion cubic meters, which is over three quarters (78%) of the reserves worldwide. In addition, the “B&R” initiative also connects East Asia and Europe, the two major energy consumption regions. The implementation of this initiative will strengthen the connection between the energy producers and consumers. Therefore, whether Chinese energy companies should continue to expand their energy investments in “B&R” countries and how to choose a location with investment advantages in such countries are both important issues, which are directly related to the gains and risks of China’s OFDI in energy sector.

In this way, what are key factors for Chinese energy companies when investing in “B&R” countries? In this paper, we emphasize that the “intimate” relations between China and “B&R” countries are important. The “intimate” relation means the lower transaction cost of FDI and thus may promote FDI flows. That is, most “B&R” countries have advantages such as similarity to Chinese culture and closer institutional distance. Therefore, these advantages have positive impacts on transnational investment by easier contracts implementation and promoting the cross-border flows of information [8–10].

However, the previous research mostly focused on the influence of bilateral geographic factors, colonial relations, and other variables, which are usually used as control variables [11–13]. Variables those represent the “intimate” relations between the two countries are ignored, for instance, the genetic distance, the number of immigrants, the number of bilateral senior leaders’ visits, and the institutional distance, etc. Such factors may even affect the bilateral OFDI in energy sector more than the traditional geographic factors. Therefore, this paper further explores to which extent the “intimate” relations between the two countries can affect China’s OFDI in energy sector, such as the genetic distance, the number of immigrants, the number of bilateral senior leaders’ visits, and the institutional distance.

Based on the data of China’s OFDI in energy sector in 133 countries from 2005 to 2014, this paper uses a gravity model to investigate the impacts of “intimate” relations on China’s OFDI in energy sector. We find that the four “intimate” variables, namely the genetic distance, the number of immigrants, the number of bilateral senior leaders’ visits, and the institutional distance, have significant effects on China’s OFDI in energy sector. Further, considering China’s “intimate” relations with countries along the “Belt and Road” and current locations of China’s OFDI, we find that China should further expand energy investment in the “B&R” countries.

We contribute to the literature in several ways. First, following the features of China’s OFDI, we re-examine the role of “intimate” relations in driving China’s OFDI in energy sector by estimating a gravity model which particularly includes variables those are not emphasized in the traditional gravity model, such as genetic distance, the number of immigrants, the number of bilateral senior leaders’ visits, and the institutional distance. Although the impact of some “intimate” variables on developed economies is acknowledged in previous research [9, 10, 14], the explanatory value of these factors for understanding OFDI from developing countries is still unknown. Moreover, this lack of knowledge of how “intimate” factors affects China’s OFDI may stands in contrast with policy deliberations in many countries those are trying to attract China’s OFDI.

Second, this paper focuses on OFDI in energy sector, which is different from the previous literature that mainly focuses on the total amount of OFDI between countries [15–18]. Energy products, such as oil and gas, are not only essential productive factors for economic development, but also important strategic materials related to international politics, regional relations, and global capital markets [7, 19]. Therefore, considering the special nature of energy investments, they are more susceptible to information flows, political and cultural communications between countries, such as “intimate” relations between countries.

The rest of this paper is arranged as follows: the second part is the literature review and hypothesis, the third part is data and empirical design, the fourth part is the empirical analysis, the fifth part is the discussion, and the last part is conclusion.

## II. Literature review and research hypotheses

Most research of the location choice of China’s OFDI includes the factors of home country and host country. In terms of home country, the institutional quality and the political risk of the home country are widely considered to be important for its OFDI. Incentive polices for capital outflows of the home country are also positive factors for OFDI [20]. When there are lot institutional constraints in the home country, companies will use foreign investments as a springboard to run businesses in host countries for their resource-rich and relatively open markets [21, 22].

For the host country, resource endowment is one of the most important factors for attracting FDI. Abundant natural resources or low labour costs will attract companies to invest in the host country [23, 24]. Natural resources, which aims to provide domestically scarce or high-cost resource for downstream production chains, are usually the goal of vertical FDI [25]. This feature is even more pronounced for the energy industry, and there are several reasons for it. First, the distribution of world energy production and demand is uneven, and it is difficult for many countries and regions to rely on their own energy reserves to meet their domestic demands. However, the sustainable energy supply is an important strategic guarantee for the operation of the economic system. Therefore, many countries seek to invest in countries with abundant energy resources for their domestic economic development and energy security, as well as China [19, 26, 27]. Second, the international market for energy products is an oligopolistic market. The price of energy commodities in international market often fluctuates sharply with the global economic situation and the futures market. In order to obtain more energy reserves and strengthen risk-resistance capabilities in the context of globalization, energy companies have to make large-scale multinational investments.

However, most literature focuses on the total volume of OFDI [15–17, 28], which obscures the features of foreign investment in the specific sector or industry. Although the driving factors of OFDI have similarities in different sectors or industries, the motivation and location choice of enterprises’ investment may be also closely related to the features of the specific sector or industry.

As a special commodity, energy products such as oil and gas are not only essential productive factors for economic development, but also important strategic assets that closely related to international politics, regional relations, and capital markets [6, 7]. Thus, the OFDI in energy sector is significantly subject to policies and institutions of the host country, the information flows and cultural communication between the host and home countries. Therefore, OFDI in energy sector is more susceptible to “intimate” relations that improve the information communication and contracts implementation between home and host countries. If the home and host country become more “intimate”, the transaction cost will be lower, which promotes the FDI flows in energy sector. However, only a few of studies investigate the important role of “intimate” relations in energy sector.

Considering the impact of information friction on the total volume of OFDI, Bénassy-Quéré et al. (2007) [29] explore the impact of institutional factors on bilateral FDI and find that bilateral FDI is negatively related to the institutional distance. The closer institutional distance will help companies of the home country to better understand the potential institutional risks and investment rules of the host country. Tong (2005) [8] uses the common language as a proxy variable to measure the racial relations between countries and discusses the impact of ethnic ties on China’s FDI inflows. Javorcik et al. (2011) [9] find that immigrations can promote bilateral FDI by enhancing cross-border information flows. Blonigen and Piger (2014) [10] use a Bayesian selection model to verify that cultural distance is an important factor in bilateral FDI.

Therefore, in this paper we focus on the “intimate” variables such as the genetic distance, the number of immigrants, the number of bilateral senior leaders’ visits, and the institutional distance, to examine whether they play important roles in cross-border energy investment.

## III. Data and empirical design

### 3.1 Data and variables

#### 3.1.1 Explained variable: China’s OFDI in energy sector

Our sample includes China’s OFDI data in energy sector in 133 host countries from 2005 to 2014, which basically covers the major developed and developing countries in the world. The data is available from the China Global Investment Tracker database released by the American Enterprise Institute and the Heritage Foundation. The explained variable is China’s annual stock of OFDI in energy sector in each country (*lnInvestment*_*it*_). The subscript *i* represents the stock of energy investment in host country *i*, and the subscript *t* represents year *t*. The investment in energy sector covers petroleum, hydropower, alternative energy, natural gas and coal. We use the stock data of China’s OFDI in energy sector each year with logarithmic transformation.

Table 1 shows the investment by Chinese energy companies in “B&R” countries from 2005 to 2014. We find that Kazakhstan, Pakistan, Russia, and Iraq are the countries with the highest FDI in energy sector by Chinese companies, each of them received over 15 billion U.S. dollars of China’s energy investment from 2005 to 2014.

**Table 1:** Investment by Chinese energy companies in “B&R” countries from 2005 to 2014.

Country	Amount (Unit: hundred million U.S. dollars)	Country	Amount (Unit: hundred million U.S. dollars)
Kazakhstan	218.1	Turkey	70.3
Pakistan	182	Turkmenistan	68
Russia	154	Egypt	55.6
Iraq	152.9	Malaysia	52.4
India	134.8	Singapore	52.2
Indonesia	122.4	The Philippines	50.3
Vietnam	93.4	Cambodia	44.8
Saudi Arabia	89.1	Ukraine	41
Iran	85	Syria	37.6
Laos	75.4	The United Arab Emirates	34.9

*Notes*: Table 1 shows the investment by Chinese energy companies in “B&R” countries from 2005 to 2014. The investment covers petroleum, hydropower, alternative energy, natural gas, and coal. The data comes from the China Global Investment Tracker database released by the American Enterprise Institute and the Heritage Foundation.

#### 3.1.2 Variables of “intimate” relations

The main explanatory variables are the ones that measure “intimate” relations between countries, which are the genetic distance (*Genedist*), the number of immigrants (*lnImmigrants*), the number of bilateral senior leaders’ visits (*Visit*), and the institutional distance (*Instdist*). The definitions and data sources of the variables are as follows.

To measure the cross-country genetic distance, we use the variable *Genedist* based on the national genetic data and weighted calculation by Spolaore and Wacziarg (2009) [14]. The variable indicates the differences in sociological and cultural characteristics (customs, values, and beliefs) of different ethnic groups. Such differences are vertically transferred between generations in the same ethnic group. Therefore, ethnic groups with similar genetic characteristics cost less in communications, which in turn reduces the cost of information communications during the cross-border FDI.

Following Javorcik et al. (2011) [9], we use the variable *lnImmigrants* to measure the immigrants between each pair of countries. The Global Bilateral Migration Database of the World Bank reports data of immigrants in the years 1960, 1970, 1980, 1990, and 2000. We use the data of immigrants from host country to China in 2000 and take the logarithmic form.

We use the variable *Visit* to measure the times of senior leaders’ visits, greetings, and meetings between the two countries each year by weighted number [30]. For the chiefs of the states between two countries, we measure it as a given weight 2, and the weight of provincial and ministerial leaders’ visits is 1. The samples mainly come from the official websites of the Ministry of Foreign Affairs of China, and we complete the samples from the public reports of CCTV (China Central Television) news with hand collected.

Institutional distance (*Instdist*) measures the institutional similarity between the two countries. We use the difference of the overall institutional quality between China and other countries to measure their institutional distance. When the two countries are close in institutional quality, the home country will have a better understand of the institutional environment and hidden rules of the host country, and thus will capture changes of risks in investment better. The data is obtained from Worldwide Governance Indicators (WGI), which contains six sub-indexes indicating a country’s institutional environment from different aspects, namely the voice and accountability, political stability, and government effectiveness, regulatory quality, rule of law, and control of corruption. We take the first-order principal components of the six sub-indexes to measure the overall institutional quality of each country followed Bénassy-Quéré et al. (2007) [29].

#### 3.1.3 Control variables

The model contains the following control variables, the geographical distance between China and other countries (*Distance*), the gross domestic products of China and other countries (*lnGDPChina*, *lnGDP*), the degree of the control of corruption in each country (*Corruption*), the geographical contiguity (*Border*) and the similarity of religious beliefs (*ComReligion*) of two countries. The specific definitions and data sources of the variables are as follows.

Geographical distance (*Distance*) measures the straight-line geographic distance between Beijing (capital of China) and the capitals of other countries. The variable *Border* measures the contiguity: If the host country is contiguous with China, it takes 1, otherwise it takes 0. The variable of religion (*ComReligion*) evaluates the similarity of religious beliefs of the two countries. The data is drawn from the CEPII database of the French Centre for International Economic Research.

We also use variables of the GDP of home and host countries to control a country’s infrastructures, public services, market size and business potentials. *lnGDPChina*_*t*_ is the annual GDP per capita of China, and *lnGDP*_*it*_ is the annual GDP per capita of each host country *i*. The data is in logarithmic form and from the World Bank.

In addition, the variable *Corruption* measures the degree of the corruption controls in each host country and the data comes from Worldwide Governance Indicators (WGI) database. Goodspeed et al. (2011) [31] show that FDI is sensitive to the governance and corruption of host country. Since the corruption may be related to the “intimate” variables and affected FDI, we need to add it as a control variable.

### 3.2 Descriptive statistics

From the descriptive statistic in Table 2, we find the mean value of China’s OFDI in energy sector (stock) is 1.56 billion dollars, which indicates that the OFDI volume by Chinese energy companies are generally large. The genetic distance, the number of immigrants, the times of bilateral senior leaders’ visits, and the institutional distance between the host country and China are quite different. At the same time, their distribution is uneven, which might influence the energy companies’ location choice of OFDI.

**Table 2:** Descriptive statistics.

**Panel A:**						
**Variable**	**Definition**			**Data Source**		
*Investment*	China’s energy OFDI to each host country			China Global Investment Tracker database		
*Genedist*	Genetic distance			Spolaore and Wacziarg (2009)		
*Immigrants*	Number of immigrants			Global Bilateral Migration Database		
*Visit*	Number of bilateral senior leaders’ visits			the Ministry of Foreign Affairs of China, and CCTV (China Central Television) news		
*Instdist*	Institutional distance			Worldwide Governance Indicators (WGI)		
*Distance*	Geographic distance, logarithm			CEPII database		
*GDPChina*	Per capita GDP of China			World Bank		
*GDP*	Per capita GDP of each host countries			World Bank		
*Border*	Border on China or not			CEPII database		
*ComReligion*	Similarity of religious belief			CEPII database		
*Corruption*	Degree of the control of corruption in each host country			Worldwide Governance Indicators (WGI) database		
**Panel B:**						
**Variable**	**Unit**	**Observations**	**Mean Value**	**Standard Deviation**	**Minimum**	**Maximum**
*Investment*	million dollars	1518	1460.7971	4593.4846	0	65560
*Genedist*	/	1518	0.0201	0.0115	0	0.0396
*Immigrants*	persons	1518	902.1719	4329.5575	0	47152
*Visit*	times	1518	2.9018	3.4542	0	21
*Instdist*	/	1518	1.6339	0.9369	0.0018	3.3694
*Distance*	/	1518	9.0161	0.5170	7.0640	9.8580
*GDPChina*	dollars	1518	4391.5323	1080.0225	2738.2056	6108.2388
*GDP*	dollars	1518	13617.6863	18869.3383	219.3562	111968.3516
*Border*	/	1518	0.0791	0.2699	0	1
*ComReligion*	/	1518	0.0055	0.0086	0	23.8560
*Corruption*	/	1518	49.3711	29.3638	0	100

*Notes*: Table 2 presents descriptive statistics of the sample, which contains the data of China’s OFDI in energy sector in 133 host countries from year 2005 to 2014. *Investment* measures China’s investment in energy sector each year, which covers petroleum, hydropower, alternative energy, natural gas and coal. *Genedist* measures the genetic distance between two countries. *Immigrants* measures the immigrants between each pair of countries. *Visit* measures the times of bilateral senior leaders’ visits. *Instdist* measures the institutional distance between each pair of countries. The data contains six sub-indexes and we take the first-order principal components of the six sub-indexes. *Distance* measures the straight-line geographic distance between capitals of two countries. *Border* measures the contiguity of two countries: If the home and host countries are contiguous, it takes 1, otherwise it takes 0. *ComReligion* measures the similarity of religious beliefs of two countries. *GDPChina* measures the annual GDP per capita of China and *GDP* measures the annual GDP per capita of each host country.

### 3.3 Empirical model

We use the gravity model to investigate the impact of “intimate” relations on China’s OFDI in energy sector, which is usually used in studying international trade and FDI flows. We choose China’s annual stock of OFDI in energy sector (*lnInvestment*) as the explained variable, and choose the genetic distance (*Genedist*), the number of immigrants (*lnImmigrants*), the times of bilateral senior leaders’ visits (*Visit*), and the institutional distance (*Instdist*) as the key explanatory variables. The empirical model is as follows:

$$lnInvestment_{it}=\beta_{0}+\beta_{1}Genedist_{it}+\beta_{2}lnImmigrants_{it}+\beta_{3}Visit_{it}+\beta_{4}Instdist_{it}+\gamma Controls_{it}+Year_{t}+\varepsilon_{it}$$ (1)

where subscript *i* refers to the *i-th* country or region, *t* represents the *t-th* year, *β*_*0*_ is the constant term, and *ε*_*it*_ is the residual term. *Controls*_*it*_ stands for the control variables. According to the gravity model, the control variables include the geographic distance of China and host countries (*Distance*), China’s annual GDP per capita (*lnGDPChina*), and the host country’s annual GDP per capita (*lnGDP*). They also include whether the two countries are contiguous (*Border*), religious similarity (*ComReligion*) and control of corruption in each host country (*Corruption*). Besides, a series of dummy variables *Year*_*t*_ are also added to control the year effect.

The key coefficients are *β*_*1*_, *β*_*2*_, *β*_*3*_, and *β*_*4*_. If *β*_*1*_*<0*, it means that the genetic distance between China and the host country has significantly negative impact on China’s OFDI in energy sector, which provides the evidence that the differences in demographic, social, and cultural aspects between the two countries do affect their cost of communications, which subsequently affects the location choice of OFDI in energy sector. Similar to *β*_*1*_, the coefficients as *β*_*2*_, *β*_*3*_, and *β*_*4*_ represent whether the number of immigrants, the times of bilateral senior leaders’ visits, and the institutional distance have significant effects on China’s OFDI in energy sector respectively.

As for the estimation strategy, we mainly use the pooled OLS regression to obtain the benchmark regression results and apply the Hausman-Taylor approach of panel data as the robustness test. Hausman-Taylor approach based on panel data is able to capture unobservable national characteristics and reduce endogenous bias caused by missing variables [32]. Therefore, we set the gravity model based on Hausman-Taylor approach as follows:

$$lnInvestment_{it}=\beta_{0}+\beta_{1}Genedist_{it}+\beta_{2}lnImmigrants_{it}+\beta_{3}Visit_{it}+\beta_{4}Instdist_{it}+\gamma Controls_{it}+Year_{t}+\mu_{i}+\varepsilon_{it}$$ (2)

where the error term is *μ*_*i*_ + *ε*_*it*_. *μ*_*i*_ is the individual effect of host country, and *ε*_*it*_ refers to other unobserved factors affected investment besides the explanatory and control variables. The rest of the model settings and the regression coefficients we focused on are the same as Eq (1).

Hausman Taylor approach is used to solve the difficulty of estimating gravity model with panel data, which provides a framework of instrumental-variable estimation method by Hausman and Taylor (1981) [32–35]. The difficulties are: First, if we estimate the gravity model of fixed effect, we cannot estimate the coefficients of variables those do not change over time, such as geographic distance and border; Second, if we estimate the gravity model of random effect, it requests that the individual effect (i.e., *μ*_*i*_) is independent of all explanatory variables, which is difficult to satisfy in practice.

Therefore, to solve the difficulty of estimating gravity model with random effect, Hausman-Taylor approach allows the individual effect to be correlated with explanatory variables. The endogenous problem caused by the correlation between individual effect and explanatory variables is solved by using instrumental variables with the information inside the model [33]. In this way, Hausman-Taylor method provides consistent parameter estimates of the gravity model under panel data, allowing both time-varying factors and time-invariant factors, when OLS or the traditional random-effects model are biased due to missing variables [32].

## IV. Empirical analysis

### 4.1 Baseline results

The results of the effect of “intimate” relations on China’s OFDI in energy sector are summarized in Table 3. Columns (1) to (3) show the results of pooled OLS, and columns (4) to (6) show the results of Hausman-Taylor model. Keeping other factors controlled, all of the four “intimate” variables, the genetic distance (*Genedist*), number of immigrants (*lnImmigrants*), times of visits by bilateral senior leaders (*Visit*), and institutional distance (*Instdist*), have significant impact on China’s OFDI in energy sector.

**Table 3:** The impact of “intimate” relations on China’s OFDI in energy sector: Baseline results.

	(1)	(2)	(3)	(4)	(5)	(6)
	OLS			Hausman-Taylor		
*Genedist*	-0.6132***	-0.8506***	-0.8457***	-1.4482***	-1.8127***	-1.6863***
	(0.1096)	(0.1120)	(0.1123)	(0.5480)	(0.4913)	(0.4370)
*lnImmigrants*	0.4850***	0.4527***	0.4503***	0.5294***	0.4865***	0.4616***
	(0.0360)	(0.0356)	(0.0357)	(0.1478)	(0.1268)	(0.1198)
*Visit*	0.2235***	0.2032***	0.2089***	0.0520***	0.0533***	0.0544***
	(0.0253)	(0.0250)	(0.0254)	(0.0194)	(0.0195)	(0.0199)
*Instdist*	-1.3947***	-0.8468***	-0.8640***	-1.5953***	-0.9577***	-1.0916***
	(0.0992)	(0.1411)	(0.1424)	(0.3040)	(0.2889)	(0.3106)
*Distance*	-1.1700***	-1.7787***	-1.7873***	-1.0361	-2.6953**	-2.2708***
	(0.1763)	(0.1957)	(0.1962)	(0.9594)	(1.0732)	(0.7056)
*lnGDPChina*	4.9029***	4.8528***	4.9624***	4.7746***	4.6418***	4.7058***
	(0.2840)	(0.2779)	(0.3962)	(0.2042)	(0.2033)	(0.2781)
*lnGDP*	0.3625***	0.6309***	0.6285***	1.3818***	1.9564***	1.9501***
	(0.0684)	(0.0823)	(0.0825)	(0.4146)	(0.3946)	(0.3831)
*Border*		2.2751***	2.2629***		4.4446***	4.0811***
		(0.3408)	(0.3415)		(1.4272)	(1.2559)
*ComReligion*		0.0472***	0.0466***		0.0626*	0.0502
		(0.0104)	(0.0105)		(0.0359)	(0.0339)
*Corruption*		-0.0175***	-0.0172***		-0.0413***	-0.0405***
		(0.0047)	(0.0047)		(0.0108)	(0.0109)
*Constant*	-54.2318***	-62.8876***	-63.9799***	-63.3973***	-83.2467***	-79.1548***
	(2.9966)	(3.1294)	(3.9120)	(11.1454)	(12.5175)	(8.6847)
*Year-effect*	No	No	Yes	No	No	Yes
*Country-effect*	No	No	No	Yes	Yes	Yes
*N*	1488	1488	1488	1488	1488	1488
*F*	150.5897	117.4665	65.1701	122.2577	87.7727	49.0104

*Notes*: Table 3 presents the estimates of the effect of “intimate” relations on China’s OFDI in energy sector. The explained variable is China’s OFDI in energy sector. The explanatory variables are introduced in section II. Standard errors are reported in parentheses below coefficient estimates. Statistical significance is indicated by *,**,*** for the 10%, 5% and 1% levels respectively.

As mentioned in section 3.3, the Hausman-Taylor approach can better avoid the bias of endogeneity. Therefore, we mainly explain the coefficients of “intimate” relations estimated by the Hausman-Taylor approach after adding all the control variables and year effects in column (6). First, we find that for every 1% of increase in a country’s genetic distance with China, China’s OFDI in energy sector will decrease by 1.69%. One of the possible explanations for this significant effect is that the genetic distance, as a measurement of cultural heterogeneity among different nations, plays an important role in bilateral economic exchanges. The cultural difference among generations across countries is fundamentally determined by the genetic difference, which is inherited from the previous generation and expressed in genetic distance. The large cultural gap between the two regions tends to reduce the bilateral economic cooperation of the two regions [14, 36].

Secondly, for every 1% more of the immigrants from the host country, China’s OFDI in energy sector for the host country will increase by 0.46%. The reason might be that the immigrants from the host country bring more information about the host country, which mitigates the information asymmetry and friction cost.

In terms of the times of visits by bilateral senior leaders (*Visit*), as it shows in column (6), indicating that for every one more time of visit by the bilateral senior leaders between China and the host country, China’s OFDI in energy sector will increase by 5.44%. This suggests that the leaders’ visit has significantly affect the location choice of China’s energy OFDI. Leaders’ visit is a direct and effective way to increase political mutual trust and build the friendship between the two countries. On the one hand, the risk of bilateral political uncertainty is reduced by the friendly transmission signals and credit guarantees. On the other hand, direct dialogues and political consultations effectively resolve the major bilateral problem of interests, enhancing the confidence of energy investment companies in long-term investment in the host country.

Finally, the institutional distance (*Instdist*) also has a significant effect on energy OFDI, every 1% decrease of the institutional distance between China and the host country will lead to 1.09% increase of China’s OFDI in energy sector. That is to say, the greater the institutional difference between the host country and China, the less China’s energy investment in the host country. One of the reasons might be that less institutional similarity leads to more difficulties for Chinese energy companies to better understand the potential institutional risks and investment rules of the host country, and consequently inhibits the OFDI.

Therefore, all of the four “intimate” variables we focus on have significant effects on China’s OFDI in energy sector. In addition, the coefficients of GDP per capita of China and host countries (*lnGDPChina*, *lnGDP*) are significant at the level of 1% and have positive effects on OFDI, indicating that the economic development of home and host countries will both promote the energy investments. Consistent with the literature, the distance between the two countries (*Distance*) and the control of corruption (*Corruption*) have significant negative impacts on China’s OFDI in energy sector, which means that the closer distance improves but the higher-level corruption discourages energy investment. In addition, the contiguousness (*Border*) and the similarity between the two countries (*ComReligion*) have significant positive impacts on China’s energy OFDI, indicating that the closer the two countries are in geography and religion, the more investment flows between the two countries.

### 4.2 Developed and developing countries

In the robustness test, we try to test whether there are different impacts of “intimacy” variables on China’s energy OFDI between developed and developing host countries. We divide the sample into two sub samples according to whether the host country was an OECD country in 1980, and then estimates the two sub samples. The results are summarized in Table 4.

**Table 4:** The impact of “intimate” relations on China’s OFDI in energy sector: Considering developing and developed countries.

	(1)	(2)	(3)	(4)
	Developing Countries		Developed Countries	
*Genedist*	-0.5382***	-0.5287***	-1.0755***	-1.0547***
	(0.1281)	(0.1284)	(0.2816)	(0.2871)
*lnImmigrants*	0.4079***	0.4043***	0.9035***	0.9180***
	(0.0390)	(0.0391)	(0.1226)	(0.1255)
*Visit*	0.2453***	0.2545***	0.0017	-0.0075
	(0.0286)	(0.0290)	(0.0496)	(0.0526)
*Instdist*	-0.9758***	-1.0034***	-2.2206	-2.3924
	(0.1459)	(0.1472)	(2.0636)	(2.3541)
*Distance*	-1.6753***	-1.6877***	-2.5320***	-2.5332***
	(0.2071)	(0.2075)	(0.6758)	(0.7094)
*lnGDPChina*	4.9386***	5.2319***	4.4394***	3.9339***
	(0.2983)	(0.4252)	(0.6448)	(0.9263)
*lnGDP*	0.4629***	0.4581***	5.6357***	5.7527***
	(0.0873)	(0.0875)	(0.7968)	(0.8343)
*Border*	2.0429***	2.0214***		
	(0.3454)	(0.3460)		
*ComReligion*	0.0407***	0.0398***	0.0321***	0.0324***
	(0.0105)	(0.0105)	(0.0103)	(0.0105)
*Corruption*	-0.0164***	-0.0159***	-0.0354	-0.0344
	(0.0048)	(0.0048)	(0.0351)	(0.0387)
*Constant*	-59.8008***	-62.4387***	-116.0512***	-112.6909***
	(3.3566)	(4.1934)	(10.4675)	(12.2410)
*Year-effect*	No	Yes	No	Yes
*N*	1278	1278	210	210
*F*	102.2301	56.8855	34.6719	18.0349

*Notes*: Table 4 presents the estimates of the effect of “intimate” relations on China’s OFDI in energy sector by distinguishing investment to developed and developing countries. The explained variable is China’s OFDI in energy sector. The explanatory variables are introduced in section II. The sample includes 133 host countries, and we classify developed countries and developing countries according to whether the host country was an OECD country in 1980. We have 19 countries as the subsample of developed countries: Australia, Austria, Belgium, Canada, Switzerland, Germany, Denmark, Spain, Finland, France, Greece, the United States, Italy, Japan, the Netherlands, Norway, New Zealand, Portugal, Sweden. The remaining 114 countries are classified as developing countries. Columns (3) and (4) show the regression results of the sub samples of developed countries, where the regression coefficient of the control variable *Border* is missing because China does not border the 19 developed countries in the subsample. Standard errors are reported in parentheses below coefficient estimates. Statistical significance is indicated by *,**,*** for the 10%, 5% and 1% levels respectively.

In Table 4, for the sub sample of developing countries, the four key explanatory variables, i.e., *Genedist*, *lnImmigrants*, *Visit* and *Instdist*, are all significant at 1% level. While for the sub sample of developed countries, the column (4) to column (6) in Table 4 show that the coefficients of *Visit* and *Instdist* are not significant. This suggests that the role of more political visits and closer institutional distance in promoting China’s energy OFDI are more effective in developing countries, but China’s energy investments in developed countries are less related to political visits and institutional factors. It shows that compared with developed countries, “intimate” relations have greater impact on China’s energy investment in developing countries. Moreover, the control of corruption does not significantly affect investment in developed countries, and it is likely that the corruption is generally controlled well in developed countries of our sample.

### 4.3 The investment motivations

In this section, we try to test whether the influence of the “intimate” variables is robust after considering investment motivations. According to the classification in the World Investment Report issued by the United Nations Conference on Trade and Development [37], the motivations of OFDI include four types: Resource-seeking, market-seeking, efficiency-seeking and created-asset-seeking. Specifically, resource-seeking OFDI refers to obtain overseas natural resources by establishing subsidiaries in the host country; Market-seeking OFDI is to expand the market or overcome the host country’s trade barriers; Efficiency-seeking OFDI refers to invest in developing countries and regions that are rich in labour and land resources with low costs; Created-asset-seeking OFDI is to obtain the advanced technology of the host country, such as advanced production processes, key manufacturing equipment or strategic management experience. In this way, the difference in host country’s resource endowment and economic development will affect the location choice of the OFDI from home country.

Considering the impacts of investment motivations, we add the variables of natural resource endowment (*Resource*), market growth (*Growth*), payment-product proportion (*Payproduct*), and technological innovation (*Tech*) of the host countries in model (1). The data of variables *Resource* and *Tech* come from World Bank, and the variable *Payproduct* is from a sub-indicator of the salary category in the Global Competitiveness Report. According to the questionnaire of the Global Competitiveness Report, the question for calculating this sub-indicator is: “In your country, how much is the salary related to your labor productivity?” “1 means completely irrelevant, 7 means greatly relevant.” We use these variables to measure different investment motivations and test whether the “intimate” relations still have a significant impact on energy OFDI after considering these investment motivations. Table 5 shows the results. The variable *Resource* is the percentage of the host country’s ore and metal exports to the country’s total merchandise exports, which represents the resource-seeking OFDI. The variable *Growth* is the annual growth rate of the GDP of the host country, which represents the market growth potentials of the host country, standing for the market-seeking OFDI. The variable *Pay-product* is the correlation of labour compensation and labour productivity in the host country. The value ranges from 1 to 7, the larger the value, the more relevant it is. We use this variable to represent the efficiency-seeking OFDI. The variable *Tech* is the proportion of the host country’s high-tech product exports to the country’s GDP, which is used to indicate the created-asset-seeking OFDI.

**Table 5:** The impact of “intimate” relations on China’s OFDI in energy sector: Considering the investment motivations.

	(1)	(2)	(3)	(4)	(5)
*Genedist*	-0.8272***	-0.8924***	-1.1421***	-1.0002***	-1.2917***
	(0.1225)	(0.1208)	(0.1402)	(0.1276)	(0.1593)
*lnImmigrants*	0.4684***	0.4158***	0.4544***	0.4334***	0.3968***
	(0.0386)	(0.0382)	(0.0430)	(0.0398)	(0.0487)
*Visit*	0.1860***	0.2207***	0.2261***	0.1973***	0.2143***
	(0.0273)	(0.0268)	(0.0306)	(0.0280)	(0.0327)
*Instdist*	-0.8294***	-0.7248***	-0.8617***	-0.8081***	-0.4636**
	(0.1651)	(0.1497)	(0.2063)	(0.1702)	(0.2343)
*Distance*	-1.8574***	-1.7642***	-2.4394***	-2.0879***	-2.2960***
	(0.2113)	(0.2040)	(0.2600)	(0.2149)	(0.2793)
*lnGDPChina*	4.5649***	4.8150***	4.4892***	4.7530***	4.3705***
	(0.4323)	(0.4234)	(0.5592)	(0.4463)	(0.6162)
*lnGDP*	0.6840***	0.7251***	0.5848***	0.6051***	0.8172***
	(0.0978)	(0.1018)	(0.1141)	(0.1021)	(0.1419)
*Border*	2.4198***	2.3025***	2.6234***	2.5795***	3.1681***
	(0.3969)	(0.3658)	(0.4385)	(0.4077)	(0.5011)
*ComReligion*	0.0519***	0.0537***	0.0520***	0.0625***	0.0895***
	(0.0119)	(0.0111)	(0.0146)	(0.0127)	(0.0173)
*Corruption*	-0.0163***	-0.0209***	-0.0125*	-0.0121**	-0.0263***
	(0.0056)	(0.0051)	(0.0067)	(0.0059)	(0.0077)
*Resource*	0.0265***				0.0337***
	(0.0051)				(0.0075)
*Growth*		-0.2303			0.6809
		(0.3865)			(0.4911)
*Payproduct*			0.4961		0.4467**
			(0.3802)		(0.2069)
*Tech*				-0.0034	-0.0234**
				(0.0089)	(0.0118)
*Constant*	-62.0600***	-63.5281***	-68.9021***	-65.6247***	-69.2131***
	(4.2693)	(4.1592)	(5.6883)	(4.4018)	(6.2752)
*Year-effect*	Yes	Yes	Yes	Yes	Yes
*N*	1268	1344	1038	1205	855
*F*	52.3560	54.7487	41.5488	47.8764	33.1005

*Notes*: Table 5 presents the estimates of the effect of “intimate” relations on China’s OFDI in energy sector by considering the impact of investment motivations. The explanatory variables are introduced in section II. Standard errors are reported in parentheses below coefficient estimates. Statistical significance is indicated by *,**,*** for the 10%, 5% and 1% levels respectively.

In Table 5, the coefficients of the “intimate” variables *Genedist*, *lnImmigrants*, *Visit* and *Instdist* are still significant, consistent with the above results. This indicates that after controlling the impacts of investment motivations, the “intimate” variables still have a significant impact on China’s OFDI in energy sector. In addition, no matter when the variable *Resource* is added separately in column (1) or added together with other three variables of investment motivations in column (5), *Resource* has a significant impact on China’s OFDI in energy sector at 1% level. This implies that China’s OFDI in energy sector is significantly motivated by resource seeking. In contrast, the three factors, the potential market growth in the host country (*Growth*), payment-product proportion (*Pay-product*), and capacity of technological innovation (*Tech*) are basically insignificant after being added separately and simultaneously in the empirical model. These factors failed to impose significant effect on China’s OFDI in energy sector. The result shows that the outward investment of China’s energy companies is insignificant in motivations as market seeking, efficiency seeking or created asset seeking.

### 4.4 The endogeneity problem

The “intimate” relation between two countries is not only an important factor affecting the FDI flows in energy sector, but also the FDI flows may affect the “intimate” relations as well. There may be a reverse causality between the “intimate” relations and energy FDI flows. Therefore, we use the System GMM model to alleviate the estimation bias caused by the endogeneity of reverse causality. Specifically, we set the variables of “intimate” relations (*Genedist*, *lnImmigrants*, *Visit*, *Instdist*) as endogenous variables and estimate their coefficients. Specifically, we set the variables of “intimate” relations (*Genedist*, *lnImmigrants*, *Visit*, *Instdist*) as endogenous variables, and add a one-period lagging variable *L*.*lnInvestment* as an explanatory variable to control the possible reverse causality.

The estimation results are shown in Table 6. The P values of AR(2) tests in columns (1)-(3) are all greater than 0.5, indicating that there is no second-order sequence correlation in the residuals. The P values of Sargan tests are all greater than 0.5, which means there is no over identification problem in the model. Therefore, the setting of the model is reasonable. The four variables of “intimate” relations all have significant effects on China’s energy investment in Table 6, which are consistent with the baseline results.

**Table 6:** The impact of “intimate” relations on China’s OFDI in energy sector: Considering the endogeneity problem.

	(1)	(2)	(3)
*L*.*lnInvestment*	0.9017***	0.8813***	0.8744***
	(0.0148)	(0.0294)	(0.0299)
*Genedist*	-0.0873*	-0.1231**	-0.1858***
	(0.0522)	(0.0597)	(0.0646)
*lnImmigrants*	0.0911***	0.1177***	0.1113***
	(0.0192)	(0.0241)	(0.0238)
*Visit*	0.0255*	0.0317**	0.0315**
	(0.0150)	(0.0156)	(0.0156)
*Instdist*	-0.2063***	-0.2636***	-0.0977
	(0.0452)	(0.0654)	(0.0808)
*Distance*		-0.2313**	-0.2939**
		(0.0964)	(0.1147)
*lnGDPChina*		0.1359	0.1841
		(0.2323)	(0.2343)
*lnGDP*		0.0290	0.0989**
		(0.0375)	(0.0484)
*Border*			0.2165
			(0.1954)
*ComReligion*			0.0126**
			(0.0057)
*Corruption*			-0.0065**
			(0.0026)
*Constant*	0.3260	-3.2550	-5.0808*
	(0.2503)	(2.3871)	(2.6902)
*Year-effect*	Yes	Yes	Yes
*Country-effect*	Yes	Yes	Yes
*N*	1338	1338	1338
*F*	1309.2577	589.6302	456.9144
*AR(1)*	[0.000]	[0.000]	[0.000]
*AR(2)*	[0.765]	[0.767]	[0.771]
*Sargan*	[1.000]	[1.000]	[1.000]

*Notes*: Table 6 presents the estimates of the effect of “intimate” relations on China’s OFDI in energy sector by distinguishing investment to developed and developing countries. The explained variable is China’s OFDI in energy sector. The explanatory variables are introduced in section II. Standard errors are reported in parentheses below coefficient estimates. Statistical significance is indicated by *,**,*** for the 10%, 5% and 1% levels respectively. The numbers in [] are *P* values of the statistical tests.

### 4.5 The financial crisis

In this section, we attempt to test that whether the impact of the “intimate” relations on China’s energy OFDI is robust when considering the 2008 financial crisis. Specifically, we divide the whole sample into two sub samples: Before (2005–2007) and after (2008–2014) the financial crisis, and then estimate them as pre crisis and post crisis sub samples.

The regression results are shown in Table 7. We find the variables of “intimate” relations (*Genedist*, *lnImmigrants*, *Visit and Instdist*) remain significant both before and after the financial crisis. However, the regression results of the two sub samples still show some differences: First, compared with results before the crisis, the absolute values of the regression coefficients of “intimate” variables are larger in the post crisis sub sample, which means that after the financial crisis, the “intimate” relations have greater impacts on China’s energy investment. Second, for the control variables, the coefficient of *Corruption* is not significant before the crisis, but the effect is significant after the crisis. The above results show that after the 2008 financial crisis, with the continuous increase of China’s foreign direct investment, the explanation power of political, cultural, and institutional factors for China’s energy investment is gradually enhanced.

**Table 7:** The impact of “intimate” relations on China’s OFDI in energy sector: Considering the financial crisis.

	(1)	(2)	(3)	(4)	(5)	(6)
	Year 2005—Year 2007			Year 2008—Year 2014		
*Genedist*	-0.2838*	-0.4092**	-0.4093**	-0.7540***	-1.0372***	-1.0369***
	(0.1573)	(0.1588)	(0.1590)	(0.1390)	(0.1421)	(0.1424)
*lnImmigrants*	0.3503***	0.3341***	0.3338***	0.5440***	0.5034***	0.5025***
	(0.0505)	(0.0492)	(0.0493)	(0.0462)	(0.0457)	(0.0459)
*Visit*	0.1353***	0.1115***	0.1120***	0.2424***	0.2262***	0.2277***
	(0.0411)	(0.0400)	(0.0404)	(0.0311)	(0.0309)	(0.0311)
*Instdist*	-0.6570***	-0.4948**	-0.4925**	-1.7036***	-1.0011***	-1.0005***
	(0.1449)	(0.2060)	(0.2075)	(0.1254)	(0.1783)	(0.1788)
*Distance*	-0.6624**	-1.3883***	-1.3887***	-1.3512***	-1.9113***	-1.9126***
	(0.2562)	(0.2800)	(0.2804)	(0.2228)	(0.2474)	(0.2480)
*lnGDPChina*	6.0823***	5.7540***	5.7516***	4.3575***	4.3856***	4.3442***
	(1.0835)	(1.0564)	(1.0579)	(0.5777)	(0.5647)	(0.7116)
*lnGDP*	0.1532	0.3655***	0.3652***	0.4522***	0.7349***	0.7340***
	(0.0960)	(0.1157)	(0.1158)	(0.0875)	(0.1047)	(0.1050)
*Border*		2.8597***	2.8595***		2.0052***	2.0017***
		(0.4825)	(0.4830)		(0.4325)	(0.4335)
*ComReligion*		0.0081	0.0082		0.0634***	0.0634***
		(0.0148)	(0.0149)		(0.0133)	(0.0133)
*Corruption*		-0.0066	-0.0067		-0.0213***	-0.0213***
		(0.0069)	(0.0070)		(0.0059)	(0.0059)
*Constant*	-56.5668***	-62.8824***	-62.8606***	-52.3127***	-61.6252***	-61.2897***
	(9.2727)	(9.0581)	(9.0709)	(5.3985)	(5.4752)	(6.5940)
*Year-effect*	No	No	Yes	No	No	Yes
*N*	447	447	447	1041	1041	1041
*F*	19.8350	18.4919	16.7736	100.0268	78.5159	52.1468

*Notes*: Table 7 presents the estimates of the effect of “intimate” relations on China’s OFDI in energy sector by considering the impact of financial crisis. The explanatory variables are introduced in section II. Standard errors are reported in parentheses below coefficient estimates. Statistical significance is indicated by *,**,*** for the 10%, 5% and 1% levels respectively.

### 4.6 Alternative variables

Here we try to test whether the results are robust when we use alternative variables of the key explanatory variables. In previous sections, we use the number of immigrants from host countries to China in year 2000 from the Global Immigration Database to measure the variable *Immigrants*. Now we replace the measurement of *Immigrants* as the number of immigrants from host countries to China in year 1990 and take logarithm of it. Another variable is *Instdist*, we use the World Freedom Index (FHI) as an alternative measurement of *Instdist*. The FHI is provided by Freedom House in the United States, which provides annual reports of political rights and civil liberties of 192 countries and regions in the world.

The regression results are summarized in Table 8. It shows that, when using alternative variables of the number of immigrants and institutional distance, the regression results are still consistent with the benchmark results, both in pooled OLS and Hausman-Taylor models. The variables *lnImmigrants* and *Visit* are significantly positive, and the variables *Genedist* and *Instdist* are significantly negative.

**Table 8:** The impact of “intimate” relations on China’s OFDI in energy sector: Using alternative explanatory variables.

	(1)	(2)	(3)	(4)	(5)	(6)
	OLS			Hausman-Taylor		
*Genedist*	-0.6482***	-0.9370***	-0.9345***	-1.5174***	-1.8756***	-1.6978***
	(0.1101)	(0.1111)	(0.1113)	(0.5511)	(0.4842)	(0.4240)
*lnImmigrants*	0.6102***	0.6031***	0.6003***	0.5089***	0.5934***	0.5402***
	(0.0458)	(0.0451)	(0.0453)	(0.1866)	(0.1609)	(0.1480)
*Visit*	0.2105***	0.1815***	0.1859***	0.0530***	0.0539***	0.0552***
	(0.0259)	(0.0253)	(0.0258)	(0.0195)	(0.0195)	(0.0196)
*Instdist*	-0.0544***	-0.0330***	-0.0331***	-0.0607***	-0.0374***	-0.0379***
	(0.0034)	(0.0046)	(0.0046)	(0.0101)	(0.0095)	(0.0095)
*Distance*	-1.1754***	-1.8988***	-1.9039***	-0.7268	-2.8956***	-2.2449***
	(0.1767)	(0.1965)	(0.1970)	(0.9119)	(1.0691)	(0.6800)
*lnGDPChina*	4.8823***	4.8589***	4.8329***	4.7033***	4.6273***	4.9347***
	(0.2855)	(0.2765)	(0.3935)	(0.2118)	(0.2046)	(0.4543)
*lnGDP*	0.1770***	0.4910***	0.4895***	1.2949***	1.7712***	1.7341***
	(0.0658)	(0.0830)	(0.0832)	(0.4418)	(0.3953)	(0.3845)
*Border*		2.4772***	2.4681***		4.6904***	4.1464***
		(0.3389)	(0.3398)		(1.4019)	(1.2145)
*ComReligion*		0.0672***	0.0670***		0.0816**	0.0684**
		(0.0104)	(0.0104)		(0.0363)	(0.0329)
*Corruption*		-0.0171***	-0.0170***		-0.0409***	-0.0418***
		(0.0045)	(0.0045)		(0.0108)	(0.0108)
*Constant*	-52.1270***	-62.9395***	-62.8437***	-58.7410***	-83.2880***	-78.5716***
	(2.9818)	(3.1073)	(3.8815)	(10.3339)	(12.2942)	(8.7919)
*Year-Effect*	No	No	Yes	No	No	Yes
*Country-effect*	No	No	No	Yes	Yes	Yes
*N*	1488	1488	1488	1488	1488	1488
*F*	147.3684	120.3769	66.6932	122.4322	88.5190	59.3902

*Notes*: Table 8 presents the estimates of the effect of “intimacy” on China’s OFDI in energy sector using the alternative explanatory variable of *lnImmigrants* and *Instdist*. The dependent variable is China’s OFDI in energy sector. The independent variables are selected in section II. Standard errors are reported in parentheses below coefficient estimates. Statistical significance is indicated by *,**,*** for the 10%, 5% and 1% levels respectively.

## V. Dominance analysis and discussion

In the above, we investigate the importance of “intimate” factors on bilateral FDI flows from the perspective of statistical significance. However, another important issue is how to determine the contribution of “intimate” relations among many explanatory variables mentioned above. The approach of dominance analysis provides an effective solution. This approach is proposed by Budescu (1993) [38] to determine the relative importance of explanatory variables in multiple regressions. It tests the relative importance of explanatory variables by testing the *R*^*2*^ values of all possible subset models. Compared with traditional methods, dominance analysis can compare all sub-models derived from the full model and predict the relative importance of the variables. Based on the comparison of all subsets of regressions, dominance analysis is one of the most effective methods for measuring the contribution of explanatory variables or explanatory-variable sets in regression models [39].

Using dominance analysis, the contribution of each “intimate” variable in the full-sample regression is shown in Table 9. Among the four “intimate” variables, the number of immigrants contributes most, with a contribution degree of 0.2141, followed by the times of bilateral senior leader visits, institutional distance, and genetic distance.

**Table 9:** Analysis of “intimate” variables based on dominance analysis.

Variables of “Intimate” Relations	Contribution	Standardized contribution	Ranking
*lnImmigrants*	0.0951	0.2141	1
*Visit*	0.0615	0.1386	2
*Instdist*	0.0414	0.0933	3
*Genedist*	0.0202	0.0456	4

*Notes*: Table 9 presents the tests of the relative importance of explanatory variables by testing the R^2^ values of all possible subset models using domain analysis.

Based on the empirical results and dominance analysis, we would like to check whether China’s OFDI in energy sector is mainly located in countries along the “Belt and Road”. From the above research, we find the “intimate” relations indeed play an important role in China’s energy OFDI. Now we want to analyse which countries are more “intimate” with China along the “Belt and Road”, in order to infer China’s future locations of energy investment.

We focus on analysing four variables, immigrations, bilateral senior leaders’ visits, institutional distance, and genetic distance, which have both significant regression coefficients and great contributions in the dominance analysis. As shown in Table 10, as for the countries of greatest number of immigrants, only 6 of the top 10 countries are “B&R” countries. While for the countries with most bilateral visits of senior leaders to China, there are also only 6 of the top 10 countries are “B&R” countries. For the countries those are closest to China in institutional distance and genetic distance, there are 6 and 5 among the top 10 economies are “B&R” countries, respectively. This indicates that China’s energy investment in “B&R” countries has not yet reached the desired level from the perspective of “intimate” relations, as the “B&R” countries only account for about half of the top 10 countries.

**Table 10:** Comparative analysis of “intimate” relations across countries.

Country	*lnImmigrants*	“B&R”	Country	*Instdist*	“B&R”
South Korea	47152		Laos	0.0370	Yes
Brazil	16246		Sudan	0.0952	
Thailand	14829	Yes	Belarus	0.1027	Yes
Indonesia	8586	Yes	Iran	0.1109	Yes
Malaysia	7278	Yes	Cuba	0.1118	
India	5767	Yes	Saudi Arabia	0.1351	Yes
Vietnam	4131	Yes	Zimbabwe	0.1996	
Australis	3578		Vietnam	0.2011	Yes
Peru	2897		Tajikistan	0.2744	Yes
Pakistan	2460	Yes	Chad	0.2795	
Country	*Visit*	“B&R”	Country	*Genedist*	“B&R”
Japan	11.4		Cameroon	0.0011	
South Korea	11.4		Canada	0.0018	
France	10.4		Mongolia	0.0021	Yes
Kazakhstan	10.3	Yes	Japan	0.0035	
India	9.8	Yes	South Korea	0.0035	
Germany	9.7		Madagascar	0.0036	
Vietnam	8.3	Yes	Indonesia	0.0047	Yes
Pakistan	8.2	Yes	Cambodia	0.0047	Yes
Cambodia	8.0	Yes	Laos	0.0047	Yes
Thailand	7.7	Yes	Malaysia	0.0047	Yes

*Notes*: Table 10 presents the top 10 countries with most immigrants to China, most bilateral senior leaders’ visits with China, least institutional distance and genetic distance with China respectively.

Therefore, there is still a lot room for energy cooperation between the “B&R” countries and China. “B&R” countries are rich in natural resources such as oil, and natural gas. The proven reserves of oil in the countries along the “Belt and Road” account for 54% of the world’s total volume, and the production of oil by oil-producing “B&R” countries account for 52% volume of the world. It is preliminarily estimated that the further new proven reserves in “B&R” countries will be no less than 50% of the total volume in the world. Regarding the natural gas, the 65 “B&R” countries and regions, with vast geographic span, involve the three major natural gas storage areas in the world, namely the Middle East, Russia, and Central Asia. According to the BP Statistical Review of World Energy (2018), as of the end of 2017, the total proven natural gas reserves of the “B&R” countries are about 155.4 trillion cubic meters, accounting for 58.3% the world’s total. The natural gas output of the “B&R” countries totalled 1.98 trillion cubic meters, over a half (57.3%) of the global natural gas output in 2017. In addition, for the economic potentials, the “B&R” countries have 44% of the world’s populations and over 50% of resources, while their assembled GDP is only one fifth of the world. These countries had an average economic growth rate of 3.13% in 2016, which had been higher than the OECD countries and the global average since 2000, indicating a tremendous potential in economic development.

However, the growth rate of investment by Chinese energy companies in “B&R” countries has slowed down, and the proportion of investment in these countries to the total investment declined from 92.26% in 2005 to 75.07% in 2017, and even the lowest to 44.19% in 2012. Therefore, China could continue to expand energy investments in “B&R” countries.

## VI. Conclusion

Based on the data of China’s OFDI in energy sector in 133 countries from 2005 to 2014, we examine the “intimate” relations on China’s OFDI in energy sector and the location choice of the investment from Chinese energy companies. The results are as follows:

First, the four “intimate” variables have significant effects on China’s OFDI in energy sector. Holding other variables fixed, for each one more bilateral senior leaders’ visit between China and the host country, China’s OFDI in energy sector for the host country will increase by 5.44%. If the genetic distance from China and host country increases by 1%, China’s OFDI in energy sector will fall by 1.69%. For every 1% increase in the institutional distance between China and host country, China’s energy OFDI will decrease by 1.09%. For every 1% increase in a country’s immigration to China, China’s energy OFDI will increase by 0.46%. After considering the impact of financial crisis and using alternative variables, the results are still robust.

Second, after distinguishing developed and developing countries, we find that compared with developed countries, “intimate” relations have greater impacts on China’s energy OFDI in developing countries. In developed countries, the number of bilateral senior leaders’ visits and institutional distance do not have significant impacts on China’s energy OFDI. Further research finds that resource seeking is the main motivation for China’s OFDI in energy sector.

Third, using dominance analysis, we find the number of immigrants contributes the most to China’s OFDI in energy sector, with a contribution of 0.2141, followed by the times of bilateral senior leaders’ visits, institutional distance and genetic distance. On this basis, combining the features of China’s “intimate” relations with “B&R” countries and the analysis of China’s OFDI locations, we find that China should further expand energy investment in “B&R” countries.

The empirical results of this paper are valuable for policy makers and entrepreneurs involved in China’s OFDI in energy sector. Our results indicate that strengthening political and cultural exchanges with host countries, especially with those developing countries, is conducive to the outward investment of energy companies. The foreign relations between the home and host countries may have a significant impact on their investment policies.

For energy companies, they need to properly handle their relations with countries rich of resources. It is also necessary to take detailed investigations on the host country, get familiar with the host country’s culture, respect local customs and labour policies, so as to ensure a successful outward investment in host countries.

## Supporting information

S1 Data (DTA)
